# Bioinformatics in crosslinking chemistry of collagen with selective cross linkers

**DOI:** 10.1186/1756-0500-4-399

**Published:** 2011-10-12

**Authors:** Radhakrishnan Narayana Swamy, A Gnanamani, Sangeetha Shanmugasamy, Ramesh Kumar Gopal, Asit Baran Mandal

**Affiliations:** 1 Microbiology Division, Central Leather Research Institute (CSIR, NewDelhi). Adyar, Chennai 600 020, Tamil Nadu, India; 2 AU-KBC, Research Centre, MIT Campus of Anna University, Chennai, India

## Abstract

**Background:**

Identifying the molecular interactions using bioinformatics tools before venturing into wet lab studies saves the energy and time considerably. The present study summarizes, molecular interactions and binding energy calculations made for major structural protein, collagen of Type I and Type III with the chosen cross-linkers, namely, coenzyme Q_10_, dopaquinone, embelin, embelin complex-1 & 2, idebenone, 5-O-methyl embelin, potassium embelate and vilangin.

**Results:**

Molecular descriptive analyses suggest, dopaquinone, embelin, idebenone, 5-O-methyl embelin, and potassium embelate display nil violations. And results of docking analyses revealed, best affinity for Type I (- 4.74 kcal/mol) and type III (-4.94 kcal/mol) collagen was with dopaquinone.

**Conclusions:**

Among the selected cross-linkers, dopaquinone, embelin, potassium embelate and 5-O-methyl embelin were the suitable cross-linkers for both Type I and Type III collagen and stabilizes the collagen at the expected level.

## Background

Collagen is the most abundant fibrillar protein in multicellular animals and the protein of importance for current scenario on development of tissue engineering materials for therapeutic applications. It has unique protein motif containing three super-coiled polyproline II helices with high percentage of imino acids. Thus, it contains repeating units of Gly-Xaa-Yaa, where, proline and 4-hydroxyproline occupy Xaa and Yaa positions respectively. Almost all prolines in the Yaa position of vertebrate collagen post-translationally modified to hydroxyproline in the presence of enzyme (prolyl-4-hydroxylase). Ever since the report of triple helical structure of collagen by Ramachandran and Kartha [1] nearly fifty years ago, there has been a considerable interest in the study of molecular properties and structure of the collagen. Molecular modeling of collagen has a long fascinating history, as Miller and Scheraga [2] reported computational model of collagen, especially evaluate the effects of specific side chains on conformation. Later Chen et al. [3] performed molecular docking to form a microfibril template based on the smith model [4]. Piez and Truzs [5] constructed three-dimensional energy-minimized model for calf-skin type-I collagen. Qi et al. [6] constructed the N-terminal te1opeptides model.

Cross linking of collagen is often necessary to improve the stability as well as resistance against enzymatic degradation. The existing cross linking agents such as formaldehyde, glutaraldehyde and epoxy compounds are all identified as cytotoxic, which limits the clinical applications and provokes scientific community to look for alternative natural cross linking/stabilizing agents. In addition, awareness on collagen modification and fibril coating in tanning has recently gained interest among Leather Chemist's. Ramasami [7] reported interaction of the collagen with small molecules like water, polyphenols and chromium (III) salt. Wu et al. [8] observed covalent interactions between glutaraldehyde and collagen. With regard to quinone tannage, Thomas and Kelly [9] reported, effect of pH on quinone fixation by collagen [9]. Stecker and Highberger [10] observed, nature of the buffer system plays an important role in collagen-quinone reactions. Theis [11] reported, collagen upon treatment with quinone, an appreciable increase in shrinkage temperature ultimately increased the structural strength of the collagen due to the bonding between polypeptide chains by means of the reaction of the reactive centers of quinone with amino groups in juxtaposition.

With regard to the bioinformatics on cross-linking chemistry of collagen, only very few reports are available collagen with polyphenols [12], and with alginic acid [13]. In order to select the suitable cross-linkers for the preparation of collagen based biomaterials, in the present study we made an attempt on bioinformatics in cross linking of selected cross linkers coenzyme Q_10_, dopaquinone, embelin, embelin complex-1 & 2, idebenone, 5-O-methyl embelin, potassium embelate (unless otherwise mentioned without potassium metal) and vilangin with collagen of both Type I and Type III. Since most of the selected cross-linkers are of from natural sources, the results of the study will find application in wet lab conditions.

## Methods

### Protein preparation

Restricting the number of repeating units is necessary in the modeling and simulation of a large macromolecule like collagen. In the present study, 24-mer collagen triple helix was constructed by Object Technology Framework (OTF) using the GENCOLLAGEN package [14]. The 24-residue long triple helix constructed corresponds to the residues 193 to 216 (2*a*1 and 1*a*2 chains) of the native type I collagen except residue 204 of the *a*1 chain, where Ala of native collagen was replaced by lysine in order to study the interaction of ligands with the side chains of basic amino acids. In case of type III collagen, all the three are identically α 1chains (3 α 1chains).

### Ligand preparation

Chemical structures of ligands such as embelin [CID no: 3218], vilangin [CID no: 417182], coenzyme Q_10 _[CID no: 5281915], idebenone [CID No: 3686], potassium embelate [CID no: 23677950], 5-O-methyl embelin [CID no: 171489] and dopaquinone [CID no: 439316] were retrieved from Pubchem compound database [15]. Unavailable three dimensional structures such as embelin complex-1 & 2 were generated using ACD/ChemSketch [16].

### Docking setup

Docking was performed using Autodock 4. Autodock combines energy evaluation through precalculated grids of affinity potential employing various search algorithms to find the suitable binding position for a ligand on a given protein [17]. Kollman united atom charges and polar hydrogens were added to the protein PDB using Autodock tools [17]. All rotatable bonds in the ligands were kept free to allow for flexible docking. Grid size was set to 40 × 40 × 40 grid points (x, y and z), with spacing between grid points kept at 0.375 Å. The Lamarckian genetic algorithm was chosen to search for the best conformers. Standard docking protocol was applied. One hundred independent docking runs were carried out for each ligand was generated by using genetic algorithm searches.

### Molecular descriptors calculation

Quantitative structure-activity relationships (QSARs) correlate the response with molecular properties of compounds under interest. Any compound to be considered as a lead must possess acceptable scores for all of the descriptors. Molinspiration [18] was used to calculate thirteen descriptors such as logP, polar surface area, molecular weight, number of atoms, number of O or N, number of OH or NH, number of rotatable bonds, volume, drug likeness (includes GPCR ligand, ion channel modulator, kinase inhibitor and nuclear receptor ligand) and number of violations to Lipinski's rule for all ligands taken for the analysis [19].

## Results and discussion

Molecular Physicochemical and the Drug-Likeness are the two properties that are important for considering a compound to become a successful drug. Table 1 depicts the International Union of Pure and Applied Chemistry (IUPAC) name and Simplified Molecular Input Line Entry Specification (SMILES) of ligands. Two dimensional structure of dopaquinone, embelin, potassium embelate and 5-O-methyl embelin also represented in additional files 1, 2, 3 and 4. A chemical nomenclature is a set of rules followed to generate systematic names for chemical compounds. IUPAC nomenclature is a universal chemical nomenclature. It is developed and kept up to date under the auspices of the International Union of Pure and Applied Chemistry (IUPAC). The simplified molecular input line entry specification (SMILES) is a specification for unambiguously describing the structure of chemical molecules using short ASCII strings. SMILES strings can be imported by most molecule editors for conversion back into two-dimensional drawings or three-dimensional models of the molecules.

**Table 1:** Ligand Molecules - IUPAC Name and SMILES

Ligand	IUPAC Name	SMILES
Coenzyme Q 10	2-[(2E,6E,10E,14E,18E,22E,26E,30E,34E)- 3,7,11,15,19,23,27,31,35,39-decamethyltetraconta-2,6,10,14,18,22,26,30,34,38-decaenyl]-5, 6-dimethoxy-3-methylcyclohexa-2,5-diene-1,4-dione	CC1=C(C(=O)C(=C(C1=O)OC)OC)CC=C(C)CCC=C(C)CCC=C(C)CCC=C(C)CCC=C(C)CCC=C (C)CCC=C(C)CCC=C(C)CCC=C(C)CCC=C(C)C
Dopaquinone	(2S)-2-amino-3-(3,4-dioxocyclohexa-1,5-dien-1-yl)propanoic acid	C1=CC(=O)C(=O)C=C1CC(C(=O)O)N
Embelin	2,5-dihydroxy-3-undecylcyclohexa-2,5-diene-1,4-dione	CCCCCCCCCCCC1=C(C(=O)C=C(C1=O)O)O
Embelin complex-1	Not available	O=C2C(CCCCCCCCCCC)=C(O)C([OH+][OH+][OH+][OH+]C=1C(CCCCCCCCCCC)=C(O)C(=O)CC=1O)=CC2O
Embelin complex-2	Not available	O=C2C=C(O)C(=O)C(CCCCCCCCCCC)=C2OO[N+](/[O-])=[O+]\[O+](OC1=CC(=O)C(O)=C(CCCCCCCCCCC)C1=O)[N+]([O-])=O
Idebenone	2-(10-hydroxydecyl)-5,6-dimethoxy-3-methylcyclohexa-2,5-diene-1,4-dione	CC1=C(C(=O)C(=C(C1=O)OC)OC)CCCCCCCCCCO
5-O-Methyl embelin	2-hydroxy-5-methoxy-3-undecylcyclohexa-2,5-diene-1,4-dione	CCCCCCCCCCCC1=C(C(=O)C=C(C1=O)OC)O
Potassium embelate	6-hydroxy-3,4-dioxo-5-undecylcyclohexa-1,5-dien-1-olate	CCCCCCCCCCCC1=C(C(=CC(=O)C1=O)[O-])O

Vilangin	2-[(2,5-dihydroxy-3,6-dioxo-4-undecylcyclohexa-1,4-dien-1-yl)methyl]-3, 6-dihydroxy-5-undecylcyclohexa-2,5-diene-1,4-dione	CCCCCCCCCCCC1=C(C(=O)C(=C(C1=O)O)CC2=C(C(=O)C(=C(C2=O)O)CCCCCCCCCCC)O)O

The rule formulated by Christopher A. Lipinski et al. [20] considered as the thumb rule thumb rule to evaluate drug likeness, or determine if a chemical compound with a certain pharmacological or biological activity has properties that would make it a likely orally active drug in humans. The rule describes molecular properties important for a drug's pharmacokinetics in the human body, including their absorption, distribution, metabolism, and excretion ("ADME"). The rule is important for drug development where a pharmacologically active lead structure is optimized step-wise for increased activity and selectivity, as well as drug-like properties as described by Lipinski's rule.

LogP (Octanol-water partition coefficient) is used as important tool in both quantitative structure-activity relationship (QSAR) studies and rational drug design as a measure of molecular hydrophobicity. Hydrophobicity affects drug absorption, bioavailability, hydrophobic drug-receptor interactions, metabolism of molecules, as well as their toxicity. LogP has become a key parameter in studies of the environmental fate of chemicals. In the present study LogP value of dopaquinone was -2.684, which indicates more hydrophilic nature, whereas LogP value of coenzyme Q _10 _was 10.509, indicates more lipophilic or hydrophobic nature. On other hand potassium embelate, idebenone, embelin and 5-O-methyl embelin demonstrated LogP value between 2.2-4.8 as shown in Table-2. LogP value less than 5 will be preferred for drug likeness property.

The preferred range of molecular weight for drug likeness property was 160-480 g/mol as reported by Tambunan and Wulandari [21]. The molecular weight of the selected cross-linkers was calculated as 195.17 g/mol (dopaquinone), 293.38 g/mol (potassium embelate), 294.39 g/mol (embelin), was 308.41 g/mol (5-O-methyl embelin) and 338.44 g/mol (idebenone). With regard to the preferred number of N, O (hydrogen bond acceptors) and OH & NH (hydrogen bond donors) 10 and or less than 10 and 5 and or less than 5 respectively compliance with the rule. From table 2, it has been observed that embelin complex-2 showed greater than 10 with respect to N and O, whereas the embelin complex-1 showed greater than 5 with respect to OH and NH. Further, the preferred number of rotatable bonds (rotb) is 15 and or less than 15, and we observed that it was greater than 15 for the cross linkers vilangin, embelin complex-1, embelin complex-2 and coenzyme Q _10 _(Table 2). The preferred number of Violations is 0, and we observed nil violations for the dopaquinone, embelin, idebenone, 5-O-methyl embelin, and potassium embelate and suggest all the five chosen ligands satisfy well with thumb rule (Table-2).

**Table 2:** The descriptor analysis helped in the identification of the better ligand

	*Coenzyme Q 10*	*Dopaquinone*	*Embelin*	*Embelin complex-1*	*Embelin complex-2*	*Idebenone*	*5-O-Methyl embelin*	*Potassium embelate without K*	*Vilangin*
**Molecular Physicochemical properties**									
Logp (Octanol-water partition coefficient)	10.509	-2.684	4.617	4.753	5.413	4.197	4.893	2.247	9.248
TPSA (Polar surface area)	52.61	97.464	74.598	172.374	227.469	72.838	63.604	77.427	149.196
No. of atoms(Number of nonhydrogen atoms)	63	14	21	44	50	24	22	21	43
M.wt (Molecular weight)	863.365	195.174	294.391	626.828	710.774	338.444	308.418	293.383	600.793
No. of ON (Number of hydrogen-bond acceptors [O and N atoms])	4	5	4	10	16	5	4	4	8
No. of OHNH (Number of hydrogen-bond donors [OH and NH groups])	0	3	2	8	2	1	1	1	4
No. of Violations (Number of Rule of 5 violations)	2	0	0	2	3	0	0	0	2
No. of rotb (Number of rotatable bonds)	31	3	10	25	27	12	11	10	22
Volume (Molecular volume)	937.819	166.513	295.208	617.365	651.349	338.282	312.736	292.466	594.584
**Drug likeness**									
GPCR ligand	-3.37	-0.21	-0.42	-0.49	-1.18	-0.22	-0.41	-0.14	-0.34
Ion channel modulator	-4.35	0.05	-0.34	-1.39	-2.47	-0.33	-0.39	-0.08	-1.07
Kinase inhibitor	-3.93	-0.84	-0.05	-0.88	-1.55	-0.21	-0.05	-0.16	-0.51

Nulcear receptor ligand	-4.04	-0.58	-0.36	-0.73	-1.83	-0.17	-0.36	-0.07	-0.52

**Figure 1:**
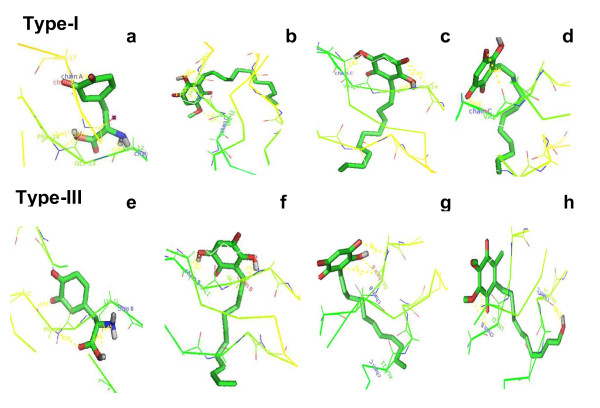
**Docking results of the four lead molecules namely dopaquinone, embelin, potassium embelate and 5-O-methyl embelin**. [Where a-e (docking results of the four lead molecules namely dopaquinone, embelin, potassium embelate and 5-O-methyl embelin) with type I collagen. Where f-i (docking results of the four lead molecules namely dopaquinone, embelin, potassium embelate and 5-O-methyl embelin) with type III collagen].

With regard to affinity and binding energy calculations, each chosen ligands displayed different affinities with the collagen types (I & III). Lead '1' dopaquinone alone showed the best affinity with both the type-I & III collagen (-4.74 kcal/mol and -4.94 kcal/mol) followed by potassium embelate, embelin, and 5-O-methyl embelin for type I collagen (Table 3 & Figure 1). However, with type III collagen, followed by dopaquinone, embelin showed the second best affinity, which was followed by potassium embelate.

**Table 3:** The binding energy helped in the identification of the better ligand

	*Coenzyme Q 10*	*Dopaquinone*	*Embelin*	*Embelin complex-1*	*Embelin complex-2*	*Idebenone*	*5-O-Methyl embelin*	*Potassium embelate without K*	*Vilangin*
**Docking results**									
Type I collagen-Lowest binding energy(kcal/mol)	0.79	-4.74	-3.6	-0.28	0.04	-3.33	-3.55	-3.78	-0.34
Type I collagen-Mean binding energy(kcal/mol)	15.69	-4.54	-3.1	0.18	0.77	-2.6	-2.82	-3.08	32.99
Type III collagen-Lowest binding energy(kcal/mol)	0.11	-4.94	-3.56	0.27	0.44	-2.93	-3.21	-3.23	1.26

Type III collagen-Mean binding energy(kcal/mol)	16.42	-4.71	-2.87	0.77	1.29	-2.42	-2.87	-2.87	11.74

With regard to the interaction sites, bond sites and bond distance, calculated based on the bioinformatics tool for the cross-linking between collagen (Type I) and four ligands (four lead molecules) as shown in table 4, emphasizes, the first lead molecule 'dopaquinone' interacts with collagen (Type I) through non-polar aminoacids (Proline and Valine) and polar aminoacid (Lysine and Asparagine). Second lead molecule 'potassium embelate' interacts with collagen (Type I) through non-polar aminoacid (Valine). Third lead molecule 'embelin' interacts with collagen (Type I) through non-polar aminoacids (Proline, Glycine and Valine). Fourth lead molecule '5-O-methyl embelin' interacts with collagen (Type I) through non-polar aminoacids (Proline and Valine) and polar aminoacid (Tyrosine).

**Table 4:** Interaction sites, bond sites and bond distance, between collagen (Type I) and four ligands.

Ligand	Interaction sites	Bond	Bond distance in(A°)
	Collagen type-I		
Dopaquinone	Eleventh residue Val of C-chain (α2)	Val C 12(Cα)-O· · ·N(Dopaquinone)	3.04
	Thirteenth residue Gly of C-chain (α2)	Gly C 13(Cα)-O· · ·H(Dopaquinone)	2.26
	Fourteenth residue Pro of C-chain (α2)	Pro C 14(Cα)-CA· · ·O(Dopaquinone)	3.07
	Seventh residue Asn of A-chain (α1)	Asn A 17(Cα)-N· · ·O(Dopaquinone)	2.79
			
Potassium embelate	Eleventh residue Val of C-chain (α2)	Val C 12(Cα)-N· · ·O(Potassium embelate)	3.03
	Eleventh residue Val of C-chain (α2)	Val C 12(Cα)-O· · ·O(Potassium embelate)	3.38
			
Embelin	Eleventh residue Val of C-chain (α2)	Val C 12(Cα)-N· · ·O(Embelin)	3.22
	Thirteenth residue Gly of C-chain (α2)	Gly C 13(Cα)-O· · ·H(Embelin)	2.74
	Fourteenth residue Pro of C-chain (α2)	Pro C 14(Cα)-N· · ·O(Embelin)	4.11
			
5-O-Methyl embelin	Eleventh residue Val of C-chain (α2)	Val C 12(Cα)-N· · ·O(5-O-Methyl embelin)	3.16
	Fourteenth residue Pro of C-chain (α2)	Pro C 14(Cα)-N· · ·O(5-O-Methyl embelin)	3.1

	Fifteenth residue Tyr of C-chain (α2)	Tyr C 15(Cα)-N· · ·O(5-O-Methyl embelin)	3.23

And with reference to type III collagen, the interaction sites, bond sites and bond distance, calculated based on the bioinformatics tool for the cross-linking of four ligands (four lead molecules) Table 5 emphasizes, the first lead molecule 'dopaquinone' interacts with collagen (Type III) through non-polar aminoacids (Proline and Alanine) and polar aminoacid (Lysine). Second lead molecule 'embelin' interacts with collagen (Type III) through non-polar aminoacid (Glycine) and polar aminoacid (Lysine). Third lead molecule 'potassium embelate' interacts with collagen (Type III) through non-polar aminoacids (Proline and Glycine). Fourth lead molecule '5-O-methyl embelin' interacts with collagen (Type III) through non-polar aminoacids (Proline) and polar aminoacid (Lysine).

**Table 5:** Interaction sites, bond sites and bond distance, between collagen (Type III) and four ligands

Ligand	Interaction sites	Bond	Bond distance in(A°)
	Collagen type-III		
Dopaquinone	Eleventh residue Val of B-chain (α1)	Val B 12(Cα)-O· · ·N(Dopaquinone)	2.6
	Eleventh residue Val of B-chain (α1)	Val B 12(Cα)-O· · ·O(Dopaquinone)	2.83
	Fourteenth residue Pro of B-chain (α1)	Pro B 14(Cα)-N· · ·O(Dopaquinone)	2.97
	Fifteenth residue Ala of B-chain (α1)	Asn B 15(Cα)-N· · ·O(Dopaquinone)	3.02
			
Embelin	Eleventh residue Lys of B-chain (α1)	Lys B 12(Cα)-N· · ·O(Embelin)	3.02
	Thirteenth residue Gly of B-chain (α1)	Gly B 13(Cα)-O· · ·O(Embelin)	2.72
			
Potassium embelate	Thirteenth residue Gly of B-chain (α1)	Gly B 13(Cα)-O· · ·O(Potassium embelate)	2.62
	Fourteenth residue Pro of B-chain (α1)	Pro C 14(Cα)-N· · ·O(Potassium embelate)	2.98
			
5-O-Methyl embelin	Eleventh residue Lys of C-chain (α1)	Lys C 12(Cα)-O· · ·O(5-O-Methyl embelin)	2.72

	Fourteenth residue Pro of C-chain (α2)	Pro C 14(Cα)-O· · ·O(5-O-Methyl embelin)	3.45

The reason behind the choice of quinone based compounds is described below. Meunier and Seyewetz [22] identified the remarkable stabilizing (tanning) potential of p-benzoquinone and after them it has been named as quinone tannage. Later, Thomas and Kelly [9] comprehensively investigated the stabilization of collagen by quinone. Wilson [23] reported benzoquinone tans/stabilizes well in alcoholic solution, and well correlated with many aspects of formaldehyde tannage. Theis [11] reported collagen treated with quinone has increased shrinkage temperature and the structural strength. Later Suparno [24] reported two salient features of quinone-tannage; viz., an increase in shrinkage temperature (Ts) (> 90°C) and high resistant to the proteolytic degradation. Further, Covington [25] also reported increase in shrinkage temperature upon treating collagen with quinone alone; however, he said the toxicity has to be considered before going for commercial use.

With regard to molecular interaction studies using bioinformatics tools, Vaidyanathan et al [26] studied the interactions of five ligands (2-Hydroxyethyl methacrylate (HEMA), Glutaraldehyde-HEMA adduct, Glyceryl dimethacrylate, Methacryloyloxyethyl maleate and Acryloyloxyethyl citraconate) with collagen and reported that steric and electrostatic complementarity interactions form the potential basis of binding between dentin adhesive ligands and type 1 collagen. Madhan et al. [27] studied stabilization of collagen by catechin, reported on the interactions of a catechin with 24-mer collagen triple helix through hydrogen bonding interaction. Mitra et al. [13] studied, thermal stabilization of collagen by alginic acid and reported on the interactions of alginic acid with 24-mer collagen triple helix through hydrogen bonding interaction with binding energy of -7.28 kcal/mol.

## Conclusion

Present study provides the molecular interaction view of quinones with both type I collagen and type III collagen, as a first eye opener on quinone and collagen interactions using bioinformatics tool. Out of nine quinone studied, dopaquinone, potassium embelate, embelin and 5-O-methyl embelin showed better affinities with both type I collagen and type III collagen. Hence dopa quinone, embelin, potassium embelate and 5-O-methyl embelin could be developed as potential cross-linking/stabilization agent of collagen preparation and found application as wound dressing sheet in clinical applications.

## Competing interests

The authors declare that they have no competing interests.

## Authors' contributions

RN, who designed and executed the experiment as part of Doctorate programme. GA & ABM Research supervisor and Director, who guided the experiment and interpretation. SS and RG who, helped to carry out the experiment and interpretation. All authors read and approved the final manuscript.

## Supplementary Material

Additional file 1 **2D structure of dopaquinone**. Figure represents the two dimensional structure of dopaquinone. Click here for file

Additional file 2 **2D structure of embelin**. Figure represents the two dimensional structure of embelin. Click here for file

Additional file 3 **2D structure of potassium embelate**. Figure represents the two dimensional structure of potassium embelate. Click here for file

Additional file 4 **2D structure of 5-O-methyl embelin**. Figure represents the two dimensional structure of 5-O-methyl embelin. Click here for file
